# A Case Series of Uncommon Sodium Disorders in Common Clinical Scenarios

**DOI:** 10.7759/cureus.35350

**Published:** 2023-02-23

**Authors:** Lakshmi Kannan, Anfal Fahim

**Affiliations:** 1 Nephrology, Pikeville Medical Center, Pikeville, USA; 2 Internal Medicine, Pikeville Medical Center, Pikeville, USA

**Keywords:** chronic kidney disease, cancer, cirrhosis, hypernatremia, hyponatremia

## Abstract

Dysnatremias or sodium disorders (either hyponatremia or hypernatremia) are the most common electrolyte problems in clinical medicine. They pose diagnostic and therapeutic challenges and have high mortality. Even mild changes in sodium levels from normal are associated with worse outcomes and considerable morbidity and mortality. We present a series of four patients with either hypo- or hypernatremia in different clinical scenarios requiring immediate treatment with close monitoring to avoid overcorrection. This case series shows that uncommon or unusual sodium disorders can happen in an otherwise usual situation.

## Introduction

Tonicity in the body is determined by sodium in the extracellular fluid (ECF) [1]. The concentration of sodium mirrors the concentration of water in the extracellular space and changes in absolute or relative water concentration change the sodium concentration. As such, total body water is maintained fairly constant by the baroreceptors, osmoreceptors, and arginine vasopressin (AVP) [2].

AVP is the most important of the three as it binds to vasopressin (V2) receptors located on the basolateral membrane of the principal cells in the renal collecting ducts. After binding, vesicles containing the aquaporin 2 water channel fuse with the luminal membrane through which the water can move from the tubular lumen to the renal medulla through aquaporins 3 and 4 located in the basolateral membrane [3]. AVP secretion is regulated by hemodynamic and non-hemodynamic changes and through osmoreceptors located in the anterior hypophysis [4].

Dysnatremia is defined as a change in sodium from the normal range of 135-145 meq/L. Hyponatremia is sodium level <135 mEq/L and hypernatremia is sodium level >145 mEq/L. Many patients remain asymptomatic (especially if a change in the plasma sodium concentration is mild and the onset is gradual), but dysnatremia can cause debilitating symptoms, such as nausea, lethargy, and seizures.

This report of four cases is aimed to show the unusual presentation of dysnatremia in otherwise usual clinical situations: hypernatremia in cirrhosis, hypernatremia in hemodialysis, hyponatremia in a patient with cancer, and hyponatremia with angiotensin-converting enzyme inhibitor (ACEi) use. 

## Case presentation

Case 1: hypernatremia in cirrhosis

A 45-year-old male with a history of traumatic brain injury, seizure disorder, and decompensated liver cirrhosis with ascites presented for progressive weakness. He was found to be confused in the emergency department (ED). His vital signs on arrival were blood pressure of 98/63 mm Hg, heart rate of 95 beats per minute, respiratory rate of 24 breaths per minute, oral temperature of 37.3 C, and oxygen saturation of 96% on 2 L nasal cannula. On physical examination, he was frail, with intermittent confusion and a distended abdomen from ascites. He appeared dehydrated on the basis of the dry oral mucosa. A serum chemistry panel showed sodium 150 mEq/L, potassium 3.6 mEq/L, chloride 108 mEq/L, bicarbonate 18 mEq/L, blood urea nitrogen (BUN) 55 mg/dL, creatinine 1.6 mg/dL, and glucose 131 mg/dL (Table 1). The patient was started on dextrose 5% in water (D5W) as he was intra-vascularly volume depleted even though he had third spacing in the form of ascites from decompensated liver cirrhosis. 

**Table 1:** Laboratory workup for the patients ESRD: end-stage renal disease; ACEi: angiotensin-converting enzyme inhibitor

Component (reference range)	Case 1	Case 2	Case 3	Case 4
	Hypernatremia in cirrhosis	Hypernatremia in an ESRD patient on dialysis.	Hyponatremia in a patient with cancer	Hyponatremia with ACEi use
Sodium (135-145 mEq/L)	150	153	116	115
Potassium (3.6-5.2 mmol/L)	3.6	3.2	3.2	3.8
Chloride (96-106 mEq/L)	108	98	112	108
Bicarbonate (22-29 mEq/L)	18	17	17	20
Blood urea nitrogen (6-24 mg/dL)	55	65	38	28
Creatinine (0.7-1.2 mg/dL)	1.6	5.7	1	1.2
Serum osmolality (275-295 mOsm/kg)	-	-	265	271
Urine osmolality (50-1200 mmol/kg)	-	-	281	246
Urine sodium (40-220 mEq/day)	-	-	57	44

Case 2: hypernatremia in a patient on dialysis

A 66-year-old male with hypertension, type II diabetes mellitus (DM), chronic obstructive pulmonary disease (COPD), and end-stage renal disease (ESRD) on hemodialysis three times a week presented to the hospital with diarrhea, fatigue, and near syncope for two days. His physical examination showed blood pressure of 155/90 mm Hg, heart rate of 68 beats per minute, oxygen saturation of 94% on room air, and a patent left upper extremity arteriovenous fistula. Blood chemistries revealed sodium at 153 mEq/L, potassium at 3.2 mEq/L, chloride at 98 mEq/L, bicarbonate at 17 mEq/L, BUN 65 mg/dL, and creatinine at 5.7 mg/dL (Table 1). The patient was taken for hemodialysis. The sodium concentration in the dialysate was 142 mEq/L. In order to avoid overcorrection, we ran D5W through a peripheral line at 40cc/hr. The patient's sodium after hemodialysis was 147 meq/L. 

Case 3: hyponatremia in a patient with cancer

A 61-year-old man with hypertension, DM, and metastatic small-cell lung cancer with new-onset seizures presented with altered behavior. On his way to the hospital, the patient had an episode of tonic-clonic seizure and was given two doses of lorazepam. His vital signs on arrival were blood pressure of 106/44 mm Hg, heart rate of 112 beats per minute, respiratory rate of 14 breaths per minute, and oxygen saturation of 94% on 2 L nasal cannula. His capillary blood glucose was 154 mg/dL. Blood chemistries revealed sodium at 116 mEq/L, potassium at 3.2 mEq/L, chloride at 112 mEq/L, bicarbonate at 17 mEq/L, BUN at 38 mg/dL, and creatinine at 1.0 mg/dL. Serum osmolality was 265 mOsm/kg, Urine osmolality was 281 mmol/kg, and urine sodium was 57. The patient was diagnosed with a syndrome of inappropriate antidiuretic hormone (SIADH) and was started on 3% hypertonic saline (Table 1).

Case 4: hyponatremia with ACEi use

An 84-year-old woman with severe dementia, hypertension, COPD, DM, and hepatocellular cancer presented to the hospital after a fall. The patient was in hospice care, found on the ground, but did not lose consciousness. When she presented to the hospital, her vital signs were blood pressure of 98/46 mm Hg, heart rate of 108 beats per minute, respiratory rate of 16 breaths per minute, and oxygen saturation of 96% on 4 L nasal cannula. Her sodium was 115 mEq/L. She was started on gentle hydration with intravenous normal saline at 60 cc/hr. Her urine osmolality was 246 mOsm/kg, urine sodium was 44 mEq/day, and serum osmolality was 271 mmol/ kg. She was placed on a fluid restriction of <1.2L/day and given furosemide 20mg twice daily. Despite that, her sodium dropped; hence, lisinopril was stopped and within a span of two days, her serum sodium normalized (Table 1).

## Discussion

In cirrhosis, hyponatremia is the most common dysnatremia. It has a prevalence of about 50%. Hypernatremia is extremely rare and data on the same are scarce. Based on a study from the Chinese Liver Transplantation Registry, 2% of patients with hepatitis B- related (HBV) cirrhosis had hypernatremia [5]. Warren et al. in 1980 [6], reviewed records of 25 patients admitted to the hospital with decompensated liver failure and found hypernatremia in 15 patients, and mortality in these patients was up by 87% compared to 60% in patients without hypernatremia. Rodes et al. in 1975 found three patients with liver cirrhosis and ascites with gastrointestinal bleeding had hypernatremia from increased BUN due to the absorption of nitrogenous compounds from the gastrointestinal tract, which resulted in osmotic diuresis [7]. In cirrhosis, hypernatremia is likely due to hypotonic fluid losses from osmotic diuresis or lactulose-induced diarrhea, and increased insensible water losses due to high ambient temperatures, pyrexia, and concomitant decreased water intake due to mental obtundation as in encephalopathy. The elderly are at greater risk. Treatment involves an expansion of plasma volume, withdrawal of diuretics, and removal of other precipitating factors [8].

In chronic kidney disease (CKD), the kidneys lose the ability to regulate water homeostasis, and the ability to maximally dilute and concentrate urine gradually declines with worsening kidney function [9]. Ultimately, urine osmolality remains constant at ~300 mOsm/L as patients reach end-stage kidney failure, regardless of the volume of water intake. As a result, patients develop hyponatremia and hypernatremia depending on other physiological factors. In a study of 655,493 United States veterans with nondialysis-dependent CKD, the prevalence of hypernatremia was 2%. This is lower than the prevalence of hyponatremia (13.5%) [10]. Pisoni et al. observed that both lower and higher serum sodium (sodium <138 and >144 mEq/L, respectively) were associated with a higher mortality risk (U-shaped association), which is different from Dialysis Outcomes and Practice Patterns Study (DOPPS I, III) and Hemodialysis (HEMO) Study [11, 12]. Hypernatremia is corrected by modifying the sodium concentration in the dialysate during hemodialysis. The rate for correction of hypernatremia is the same as in any other patient (0.5 mEq/L per hour and, at most, drop 10-12 mEq/L in 24 hours) [13].

In patients with malignancy, the event of hyponatremia/inappropriate release of antidiuretic hormone (ADH) from malignant cells is a rare phenomenon. Only a few cases of hyponatremia have been reported in cancers other than lung, such as hyponatremia after chemotherapy for acute myeloid leukemia [14], hyponatremia after radiation therapy for bladder cancer, and two cases of hyponatremia after chemotherapy for squamous cell head and neck cancer [15]. Hyponatremia develops as a result of excessive water relative to the sodium concentration and, in some cases, due to the inability of the kidneys to eliminate water in trying to keep up with the intake or due to tumor cells secreting ectopic ADH [16]. In patients receiving chemotherapy, drugs that can increase ADH production include vinca alkaloids (vincristine, vinblastine), platinum compounds (cisplatin, carboplatin), alkylating agents (cyclophosphamide, ifosfamide), and others such as methotrexate, and drugs that enhance ADH action include cyclophosphamide [17].

Medications (ACEi) most commonly associated with SIADH are captopril, enalapril, and lisinopril [18]. The possible mechanisms include blocking of angiotensin I to angiotensin II in the peripheral circulation causing excess angiotensin I to enter the brain where it is converted to angiotensin II, enhancing ADH secretion from the hypothalamus, confounding factors such as taking thiazide diuretics or selective serotonin reuptake inhibitors (SSRIs) concurrently, or presence of congestive heart failure, and beer potomania, and increased thirst with increased ADH. Asymptomatic patients with euvolemic or hypervolemic hyponatremia are usually managed initially by fluid restriction followed by discontinuation of drugs causing SIADH [18]. When SIADH is caused by a tumor, pharmacological treatment should be avoided initially because successful treatment of the malignancy may reduce the inappropriate ADH secretion. However, if pharmacological treatment is necessary, a vasopressin receptor antagonist is the drug of choice as patients can get platinum-based regimens, and these agents may mitigate the symptoms associated with hyponatremia [19].

## Conclusions

Dysnatremias occur frequently in hospitalized patients and are associated with adverse outcomes. Our case series aims to show this in different clinical scenarios. Despite their frequent occurrence, management of water and sodium balance disorders remains elusive. It is important to note that when interpreting a case of dysnatremia, patient characteristics should be considered every step of the way, and frequent measurements of plasma sodium concentration are imperative to avoid overcorrection as it could lead to osmotic demyelination.
